# Safety and Efficacy of Vancomycin–Gentamicin PMMA Bead Pouch Therapy in the Management of Pyogenic Soft Tissue Infections of the Extremities: A Case Series of 19 Patients

**DOI:** 10.3390/antibiotics15040352

**Published:** 2026-03-29

**Authors:** Stavros Goumenos, Sebastian Meller, Konstantinos Dimas, Ioannis Trikoupis, Sokratis Varitimidis, Charalampos Zalavras, Vasileios Kontogeorgakos

**Affiliations:** 1 Charité-Universitätsmedizin Berlin, Corporate Member of Freie Universität Berlin, Humboldt-Universität zu Berlin and Berlin Institute of Health, Center for Musculoskeletal Surgery (CMSC), Campus Virchow Klinikum, 13353 Berlin, Germany; stgoumenos@gmail.com; 2 Department of Pharmacology, University of Thessaly, 41500 Larissa, Greece; kdimas@med.uth.gr; 3 The Royal Orthopedic Hospital, NHS Foundation Trust, Birmingham B31 2AP, UK; giannistrikoupis@gmail.com; 4 Department of Orthopaedic Surgery and Traumatology, ATTIKON University General Hospital, National and Kapodistrian University of Athens, 12426 Athens, Greece; vaskonto@gmail.com; 5 Department of Orthopaedic Surgery and Traumatology, University General Hospital of Larissa, University of Thessaly, 41110 Larissa, Greece; svaritimidis@ortho-uth.org; 6 Department of Orthopedic Surgery, University of Southern California, Los Angeles, CA 90007, USA; zalavras@usc.edu

**Keywords:** soft tissue infection, beads, vancomycin, PMMA, local antibiotic delivery

## Abstract

**Objective**: The aim of this case series was to investigate the safety and efficacy of vancomycin–gentamicin embedded PMMA beads (VGPB) in the setting of acute pyogenic soft tissue infections (STIs) of the extremities. **Materials and Methods**: A retrospective study of 19 cases diagnosed with pyogenic STIs of the lower or upper extremity in two academic institutions was conducted between January 2017 and December 2023. All patients underwent surgical debridement, systemic antibiotics and intrawound deposition of vancomycin and gentamicin embedded cement beads (2 g of vancomycin plus 1 g of gentamicin diluted in 40 g of PMMA). Upon second look (4th–7th day post-index surgery) the cement beads were removed, serum samples from the surgical site of infection and from peripheral blood were obtained and the concentration of eluted vancomycin and gentamicin was measured. Furthermore, the white blood cell count (WBC), C reactive protein serum levels (CRP) and erythrocyte sedimentation rate (ESR) were measured before the surgical debridement and after the end of the bead therapy. All patients were reevaluated after discharge with a mean follow-up of 4.4 years (range, 1 to 7.6). **Results**: Wound vancomycin and gentamicin levels were significantly higher than those measured in the serum (34.01 ± 4.47 μg/mL versus 11.96 ± 2.79 μg/mL, *p* < 0.001 and 5.75 ± 1.22 μg/mL versus 0.51 ± 0.14 μg/mL, *p* < 0.001 respectively). Serum vancomycin and gentamicin concentrations were below the level of toxicity and no adverse events related to antibiotic-embedded bead treatment were documented. Serum WBC, ESR and CRP levels before debridement (13,446 ± 935.7 c/μL, 42.3 ± 18.7 mm/h and 113.9 ± 20.26 mg/L respectively) were significantly higher than those after the end of treatment (7889 ± 1203.6 c/μL, *p* < 0.001; 30.3 ± 9.14 mm/h, *p* = 0.017; and 22.7 ± 6.68 mg/L, *p* < 0.001 respectively). Two cases (10.5%) had a local recurrence of their STIs. Both of them relapsed within 4 months after their treatment and both had Gram-negative pathogens. **Conclusions**: Vancomycin–gentamicin PMMA bead pouch therapy appears to be a safe and effective adjuvant treatment for pyogenic soft tissue infections, offering high local antibiotic availability without systemic adverse effects.

## 1. Introduction

After the introduction of the concept of antibiotic-impregnated acrylic bone cementing as a promising means of preventing periprosthetic joint infection (PJI) after joint replacement surgery [1,2], this technique has gained enormous ground and is now being used by orthopedic surgeons around the world not only as a prophylaxis during primary cemented total joint arthroplasty, but also as an indispensable part of the single or two-stage surgical management of PJI and osteomyelitis [3,4].

In terms of a two-stage approach for the management of bone and joint infections, antibiotic-loaded polymethylmethacrylate bone cement (PMMA) has been widely used in the form of static/articulating spacers, cement blocks or entangled beads in order to provide a potent and cost-effective source for high-concentration local antibiotic delivery, but also to temporarily manage any dead space due to the bone and soft tissue defects occurring after the initial debridement [3,4,5,6,7]. With special reference to the high elution kinetics of various antibiotics from the PMMA bed and their potential for systemic host toxicity, there seems to be some disparity in the literature, as many authors support a high incidence of serious adverse events, like acute kidney injury, when on the other hand numerous studies do not report elevated toxic concentrations of the eluted antimicrobials in the bloodstream [8,9].

Further addressing the critical role of the surrounding soft tissues to the perpetuation of chronic osteomyelitis of the foot, researchers have demonstrated favorable clinical results after antibiotic-loaded bead therapy applied to chronic musculoskeletal infections of the foot and ankle [10,11].

However, whereas there are plenty of studies investigating the use of local antibiotic delivery compounds in the management of open fractures, PJI and chronic osteomyelitis with concomitant surrounding soft tissue compromise, there is little data regarding similar protocols implemented solely for soft tissue infections, which also often end up requiring multiple surgical debridements and prolonged antibiotic therapy [12].

The aim of this case series was to demonstrate the safety and efficacy of vancomycin–gentamicin embedded PMMA beads (VGPB) in the setting of acute pyogenic soft tissue infections (STIs) of the extremities and to evaluate the local and systemic bioavailability of vancomycin and gentamicin eluted from the VGPB as an adjuvant method to the surgical management of STIs.

## 2. Materials and Methods

A retrospective analysis of prospectively collected data from 19 patients treated for pyogenic soft tissue infection of the upper and lower extremities was conducted between January 2017 and December 2023 at two academic level I centers. The study was conducted according to the guidelines of the Declaration of Helsinki, and approved by the Institutional Review Board (IRB Application Number 111-08/02/2024).

**Figure 1:**
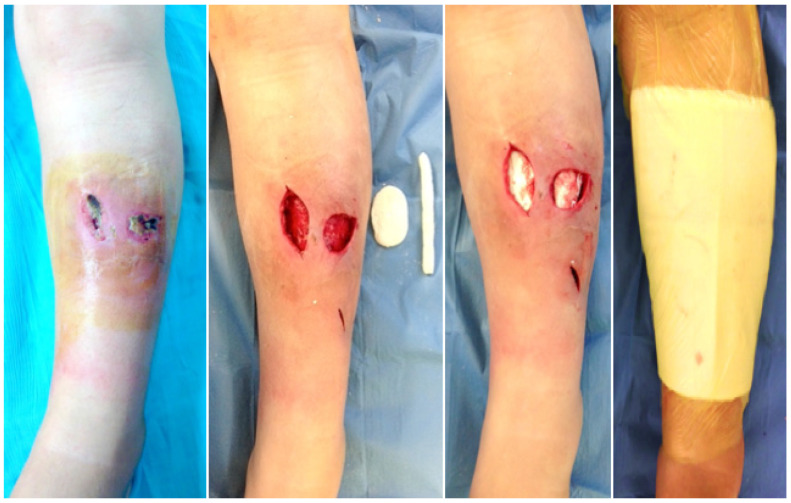
Case 1: Acute pyogenic soft tissue infection of anterolateral area of the tibia caused by *Staphylococcus aureus*. Surgical management with debridement and placement of vancomycin–gentamicin embedded PMMA beads.

**Figure 2:**
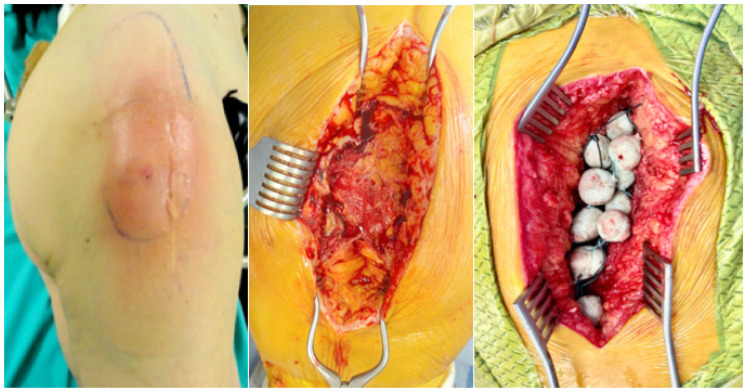
Case 2: Acute polymicrobial pyogenic soft tissue infection of the area of greater trochanter of the hip caused by Group B *Streptococci* and *Escherichia coli*. Surgical management with debridement and placement of vancomycin–gentamicin embedded PMMA bead pouch.

As for demographics, 11 patients were male and 8 were female. The mean ± standard error of age, Body Mass Index (BMI) and Charlson Comorbidity Index (CCI) were 61.3 ± 4.2, 26.7 ± 1.6 and 5.3 ± 0.6 respectively. The affected anatomical sites were: around or below the knee (9/19 patients) (Figure 1), around the hip (6/19 patients) (Figure 2), forearm (3/19 patients) and around the shoulder (one patient). Bone involvement in the infection was excluded based on clinical, radiologic and intraoperative findings. 

All 19 patients received the same treatment protocol, consisting of immediate aggressive surgical debridement of pus and necrotic tissue, deep tissue samples for microbiology, intrawound deposition of antibiotic embedded bead pouch (2 g of vancomycin plus 1 g of gentamicin diluted in 40 g of PMMA) and wound closure. Briefly, vancomycin was thoroughly mixed by hand with Refobacin-Palacos^®^ powder, which already contains gentamicin. After rigorous mixing, the cement’s liquid component was added. The cement dough was then molded into beads with large elution surface. Gentamicin and vancomycin were chosen due to their broad antimicrobial spectrum, their high PMMA elution kinetics and their known synergistic bactericidal effect [13]. All of the patients were empirically treated with a 2nd-generation cephalosporin in combination with clindamycin, administered intravenously until tissue culture results were obtained. After an average of 5.8 days (range, 4 to 7) post-index surgery, all patients underwent a second surgical debridement and VGPB removal or replacement depending on their general condition and on the local soft tissue status. Serum samples from the surgical site of infection and from peripheral blood were obtained and concentrations of eluted vancomycin and gentamicin were measured in all samples. In terms of laboratory parameters monitored, White Blood Count (WBC), Erythrocyte Sedimentation Rate (ESR) and C-reactive protein (CRP) serum levels were measured before the surgical debridement and after the end of the bead therapy. Once the implicated pathogens were identified, antimicrobial treatment was modified to a pathogen-specific regimen after infectious disease consultation. All patients were reevaluated 1 month after discharge and monitored for an average follow-up duration of 4.4 years (range, 1 to 7.6).

## 3. Statistical Analysis

Continuous variables, including demographics and laboratory parameters, were expressed as mean ± standard error with the range in parentheses. Categorical variables, including cure rates, were expressed as percentages and rates within groups. Correlations between groups were performed using the Mann–Whitney U test for eluted vancomycin and gentamicin levels and the Wilcoxon-rank test for the paired samples of WBC, ESR and CRP levels prior and after bead therapy. Differences in *p*-value < 0.05 were considered statistically significant. All data analysis was performed using the standardized SPSS software v.29 (SPSS^®^, Inc., Chicago, IL, USA).

## 4. Results

The overall cure rate in our case series was 89.5%. Two out of 19 STIs (10.5%) locally relapsed during their follow-up (2 months and 4 months after VGPB therapy respectively). The first one underwent extensive surgical debridement and remains free from signs and symptoms of infection until present. The second patient underwent below-knee amputation surgery. Both of those patients had known risk factors for infection recurrence (diabetes and peripheral vascular disease) and both had severe pyogenic soft tissue infiltration caused by Gram-negative pathogens (*Escherichia coli* and *Klebsiella pneumoniae* plus *Enterococcus faecalis* polymicrobial infection, respectively).

Attempting to better interpret the success rate of this method, we conducted a retrospective database search of 2202 patients who underwent standard debridement and received systemic antibiotics for their pyogenic soft tissue infections, without any antibiotic-impregnated PMMA beads in their wounds in our institutions. Out of the 101 patients with pyogenic soft tissue infections of the extremities requiring debridement twice, 10 of them (10.9%) had a recurrence of their infection, and thus required further wound revision surgery. The overall success of double soft tissue debridement was found to be similar to double debridement plus bead pouch treatment (10.9 versus 10.5, *p* = 0.97).

**Figure 3:**
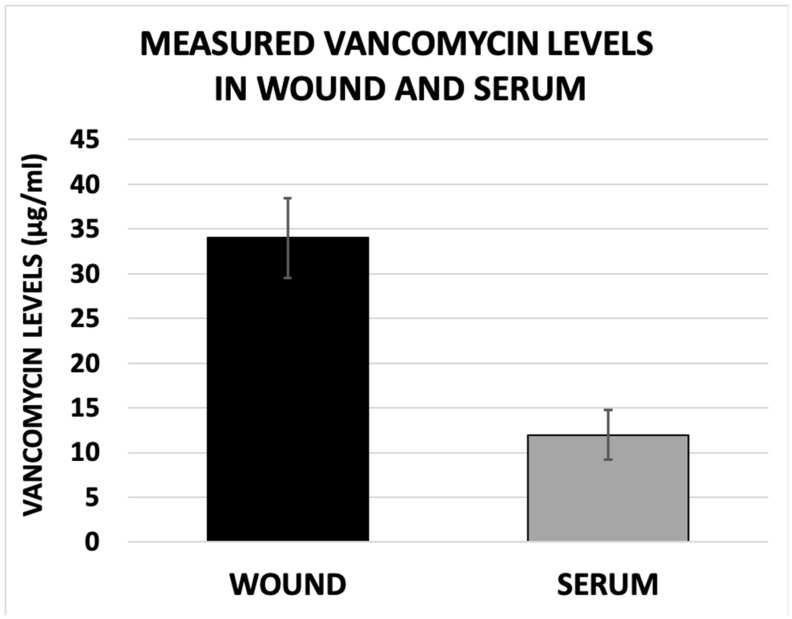
**Measured levels of eluted vancomycin in surgical site and serum**. Measured concentration of vancomycin was expressed as mean ± standard error. Statistically significant difference was found between the measured levels of eluted vancomycin from the PMMA bead pouch at surgical site and those measured in the bloodstream (34.01 ± 4.47 μg/mL versus 11.96 ± 2.79 μg/mL, *p* < 0.001).

Wound vancomycin levels (34.01 ± 4.47 μg/mL) were found to be significantly higher than those measured in the serum (11.96 ± 2.79 μg/mL, *p* < 0.001) (Figure 3).

**Figure 4:**
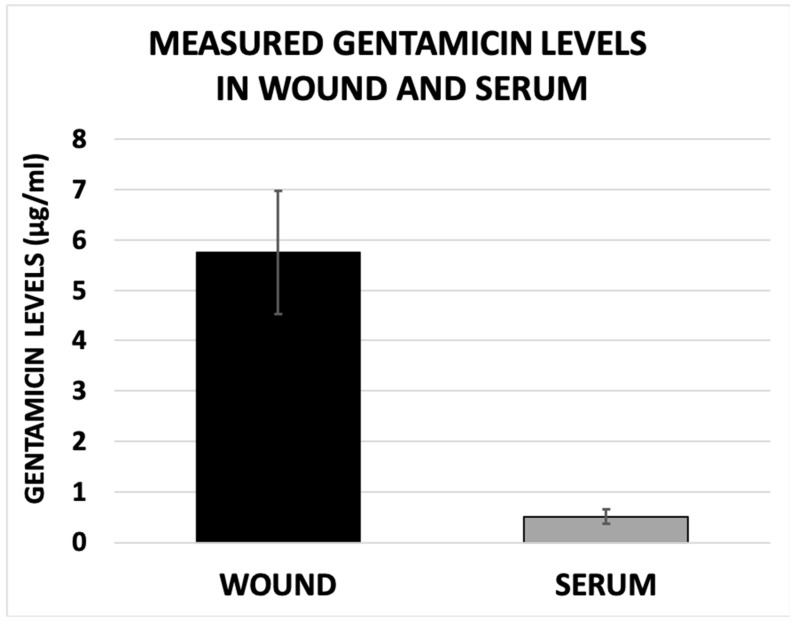
**Measured levels of eluted gentamicin in surgical site and serum**. Measured concentration of gentamicin was expressed as mean ± standard error. Statistically significant difference was found between the measured levels of eluted gentamicin from the PMMA bead pouch at surgical site and those measured in the bloodstream (5.75 ± 1.22 μg/mL versus 0.51 ± 0.14 μg/mL, *p* < 0.001).

Wound gentamicin levels (5.75 ± 1.22 μg/mL) were also found to be significantly higher than those measured in the serum (0.51± 0.14 μg/mL, *p* < 0.001) (Figure 4).

Concentrations of wound vancomycin and gentamicin were well above the MICs for all detected pathogens. Notably, serum vancomycin and gentamicin concentrations were found to be below the level of toxicity in all patients and all markers of liver and renal function were within the normal range throughout the entire treatment. No adverse events related to antibiotic-embedded bead treatment were documented.

Based on the results from the intraoperative tissue cultures, Gram (+) cocci (*Staphylococcus aureus*, Group B *Streptococci* and *Enterococci* species) were grown in 9/19 patient samples (47.4%), whereas 6/19 patients (31.6%) had polymicrobial infections, as in at least two different microorganisms implicated. The remaining STIs were caused by Gram-negative microorganisms (*Escherichia coli*, *Klebsiella oxytoca*, *Acinetobacter baumannii*, *Pseudomonas aeruginosa* and *Enterobacter*) and anaerobes.

In 7 out of 19 patients (36.8%) the VGPB therapy was prolonged for a second week after bead pouch replacement based on clinical and laboratory findings. The average duration of therapy in our case series was 11.3 ± 1.95 days.

Serum WBCs before debridement were significantly higher than those after the end of VGPB treatment (13,446 ± 935.7 c/μL versus 7889 ± 1203.6 c/μL, *p* < 0.001). The same differences were found in ESR and CRP levels as well (42.3 ± 18.7 mm/h and 113.9 ± 20.26 mg/L versus 30.3 ± 9.14 mm/h, *p* = 0.017; and 22.7 ± 6.68 mg/L, *p* < 0.001 respectively).

## 5. Discussion

The aim of our study was to investigate the efficacy and safety of vancomycin–gentamicin embedded PMMA bead pouch as part of the surgical management of acute pyogenic soft tissue infections of the extremities.

The use of antibiotic-impregnated bone cement has become a standard of practice in terms of the two-stage surgical management of chronic osteomyelitis with bone and soft tissue deficits, open fractures with a high degree of contamination and multi-stage revision for cases with septic or aseptic total joint arthroplasties [7,12,14]. Many orthopedic surgeons even prefer the use of antibiotic-embedded PMMA cement in their primary reconstruction cases as well [3]. Either molded in the form of entangled beads or in the form of a joint or diaphyseal spacer, these antibiotic-loaded constructs offer a dual therapeutic role. Firstly, they aid in the coverage of the dead space created by the infected bone and soft tissue debridement, which tends to minimize the postoperative effusion and hematoma at the surgical bed [12,15]. Secondly, based on the pathogen’s antibiogram sensitivities the polymethylmethacrylate component (PMMA) can be loaded with various individually selected antibiotic combinations, which are then gradually released directly into the site of infection, in such high concentrations that vastly exceed the minimum inhibitory or bactericidal concentrations required to eradicate the causative pathogen. This is of paramount importance, especially when there is a multi-drug-resistant biofilm-forming pathogen implicated, for the penetration of which very high levels of antibiotics are needed, which could not be administered via the intravenous route [7,16].

For our case series we chose the combination of vancomycin, which is a bactericidal glycopeptide mainly targeting Gram (+) cocci, plus gentamicin, which is an aminoglycoside bactericidal for both Gram (+) and (−) bacteriae. The high efficacy of this popular combination is well-known and has been extensively reported and demonstrated in the literature [5,13]. Apart from the broad microbiological spectrum of this regimen, a very effective synergistic effect has been described when these agents are being used in combination, offering even better odds for infection eradication [17]. Tobramycin seems to exert a similar effect, also displaying high elution concentrations from PMMA whilst offering the same antimicrobial spectrum. Hence, it is widely popular in the USA for PMMA impregnation in combination with vancomycin [18]. However, numerous research studies have emerged utilizing various and equally effective antimicrobial combinations and studying their release patterns and effectiveness in local infection control in vivo. Some of these include the combinations of daptomycin plus tobramycin [19], clindamycin plus gentamicin [20], vancomycin plus aztreonam [21] or meropenem [12], and colistin plus tobramycin [22], all exhibiting promising results in terms of elution kinetics and clinical outcome [16]. Seeley SK et al. demonstrated the wide amplification of the elution kinetics of tobramycin from PMMA showing the superiority of numerous, small, elliptically shaped cement beads in the local antibiotic diffusion rate [23].

The adjuvant use of VGPB therapy in our case series seemed to effectively control pyogenic soft tissue infections requiring one or two surgical debridements, due to the high local antibiotic concentrations achieved, which can more efficiently penetrate the biofilm formed on the surrounding soft tissues. Numerous recent research studies have demonstrated the ability of pathogens like *Staphylococci* and *Streptococci*, isolated from patients with difficult-to-treat skin and soft tissue infections, to display a high-virulence phenotype and form a mature biofilm in vitro [24,25], as well as in vivo [26], which enables them to evade host immune cells. This elicits the notion that biofilm-forming pathogens do not only form a mature biofilm on hardware implants and devascularized tissue, but also on the surrounding infected soft tissue surfaces and within microabscesses [27].

Antibiotic-loaded beads have also been used in many case series as part of the surgical treatment of chronic bone and soft tissue infections of the foot and ankle, most often in the setting of diabetic neuropathy, with favorable clinical outcomes [10,11,28]. Gorvetzian JW et al., in their retrospective study, included patients with persistent soft tissue infections who had undergone antibiotic bead-assisted surgery using absorbable beads. They concluded that the adjuvant use of beads was associated with significantly decreased septic and all-cause reoperations for chronic and infected wounds [29]. Stone PA et al. reached a similar conclusion after treating 40 patients with extracavitary vascular surgical site infections with debridement surgery and antibiotic-loaded PMMA beads displaying a cure rate of 80% in their cohort [30]. The involvement of the complicated surrounding soft tissues plays a critical role in the perpetuation of the chronic infection and the installation of a mature biofilm, which then fuels the vicious cycle of delayed wound closure and dehiscence [31]. Opposing a multi-stage approach regarding the soft tissue reconstruction of chronic diabetic ulcers, there are a few studies that propose a single-stage surgical debridement and simultaneous wound closure using meshed skin grafts [32]. In any case though, delayed wound closure has been associated with a higher rate of postoperative infection and complications versus an early closure (<72 h) [33].

The surrounding soft tissues also seem to be the major problem to be addressed in the setting of trauma. It is known that open fractures are considered to be contaminated until proven otherwise and therefore immediate stabilization and the administration of intravenous antibiotics is required with or without the adjuvant use of antibiotic-loaded cement beads onto the bed of the injury. The condition of the soft tissue envelope dictates the surgical plan for early wound closure or delayed closure after additional debridements with the use of a flap or the utilization of a negative-pressure wound device [34,35]. When it comes to the issue of applying local antibiotic carriers and PMMA beads embedded with antimicrobials on the wound of a contaminated open fracture, a meta-analysis from Morgenstern M et al. suggests a risk reduction in fracture-related infection of 12% if additional local antibiotics are given prophylactically for open-limb fractures [36]. In a retrospective cohort of 73 lower extremities requiring soft-tissue coverage after trauma, Burtt KE et al. compared the beneficial effect of antibiotic beads with negative pressure wound therapy (NPWT) and they found that the intrawound deposition of beads was associated with a decreased risk of infection, but also surgical complications when compared to NPWT [37]. Patterson JT et al. reached the same conclusion after analyzing 42 Gustilo–Anderson type IIIB tibia fractures [38]. Finally, the antibiotic bead pouch technique seems to be a valuable, uncomplicated, and cost-effective option for temporary soft tissue coverage, according to Rupp M et al. It facilitates the elution of high concentrations of the impregnated antimicrobials directly onto the affected site, effectively reducing bacterial load and eradicating biofilm infections—although the authors did not extend their indications to solely pyogenic soft tissue infections of the extremities, as in our case series [12].

Last but not least, aiming to investigate the success rate of this method compared to the standard multiple soft tissue debridement, we conducted an extended retrospective database search of 2.202 patients who underwent standard debridement and received systemic antibiotics for their pyogenic soft tissue infections, without any antibiotic-impregnated PMMA beads in their wounds, in our institutions. Taking into account that at least two debridement surgeries were estimated to be required for those severely infected cases, an arbitrary comparison between those two groups was conducted (double soft tissue debridement versus double debridement plus bead pouch treatment). Although the difference in infection recurrence rates were statistically similar between those (10.9 versus 10.5, *p* = 0.97), this could admittedly be attributed to institutional predisposition to utilize local antibiotic carriers, like PMMA beads, in those cases deemed likely to require multiple debridements or likely to involve multi-resistant pathogens or entailing significant dead space management.

The strengths of our study included the narrow and highly representative case series, which was very specific to acute pyogenic infections of soft tissues of the upper and lower extremities without obvious bone involvement. All patients were surgically managed by the same surgical team. Also, even though the analysis was conducted in a retrospective manner, all patient data were collected prospectively and the minimum follow-up duration of one year ensures that all treatment failures were well accounted for. Furthermore, we chose to focus on the vancomycin and gentamicin concentrations eluted 4–5 days after the bead pouch placement, when most research studies in the literature concentrate on the local antibiotic levels in the first postoperative days. On the other hand, we acknowledge that some major limitations of our case series were the small number of patients and the lack of a control group. Therefore, bigger prospective case–control studies with a larger sample of patients are needed in order to more thoroughly investigate the potential superiority of this method for the management of surgical soft tissue infections compared to standard debridement and systemic antibiotic administration.

## 6. Summary

The adjuvant use of vancomycin and gentamicin impregnated PMMA beads in the surgical management of pyogenic soft tissue infections of the extremities was demonstrated to have satisfactory efficacy in terms of clinical outcome, while at the same time being completely safe in terms of drug-related adverse events in our case series. The antibiotic concentrations released from the bead pouch were high in the surgical bed and significantly lower in the bloodstream, showing a well-tolerated system for local antibiotic delivery. The use of vancomycin–gentamicin PMMA bead pouches offers a simple and cost-effective strategy to deliver high local antibiotic concentrations at the infection site, especially in patients scheduled for a second-look procedure.

## Data Availability

Data is available upon request.
